# Molecular components of the circadian clock regulate HIV-1 replication

**DOI:** 10.1016/j.isci.2023.107007

**Published:** 2023-05-29

**Authors:** Helene Borrmann, Görkem Ulkar, Anna E. Kliszczak, Dini Ismed, Mirjam Schilling, Andrea Magri, James M. Harris, Peter Balfe, Sridhar Vasudevan, Persephone Borrow, Xiaodong Zhuang, Jane A. McKeating

**Affiliations:** 1 Nuffield Department of Clinical Medicine, University of Oxford, Oxford, UK; 2 Department of Pharmacology, University of Oxford, Oxford, UK; 3 Chinese Academy of Medical Sciences Oxford Institute, University of Oxford, Oxford, UK

**Keywords:** Virology, Biological sciences, Molecular biology, Bioinformatics

## Abstract

Human immunodeficiency virus 1 (HIV-1) causes major health burdens worldwide and still lacks curative therapies and vaccines. Circadian rhythms are endogenous daily oscillations that coordinate an organism’s response to its environment and invading pathogens. Peripheral viral loads of HIV-1 infected patients show diurnal variation; however, the underlying mechanisms remain unknown. Here, we demonstrate a role for the cell-intrinsic clock to regulate rhythmic HIV-1 replication in circadian-synchronized systems. Silencing the circadian activator *Bmal1* abolishes this phenotype, and we observe BMAL1 binding to the HIV-1 promoter. Importantly, we show differential binding of the nuclear receptors REV-ERB and ROR to the HIV-long terminal repeat at different circadian times, demonstrating a dynamic interplay in time-of-day regulation of HIV-1 transcription. Bioinformatic analysis shows circadian regulation of host factors that control HIV-1 replication, providing an additional mechanism for rhythmic viral replication. This study increases our understanding of the circadian regulation of HIV-1, which can ultimately inform new therapies.

## Highlights

• HIV-1 replication in circadian-synchronized cells is rhythmic
• ROR inhibitors suppress rhythmic HIV-1 replication
• Circadian factors regulate HIV-1 transcription via binding to the viral promoter
• HIV-1 host factors are under circadian control

## Graphical abstract

**Figure:**
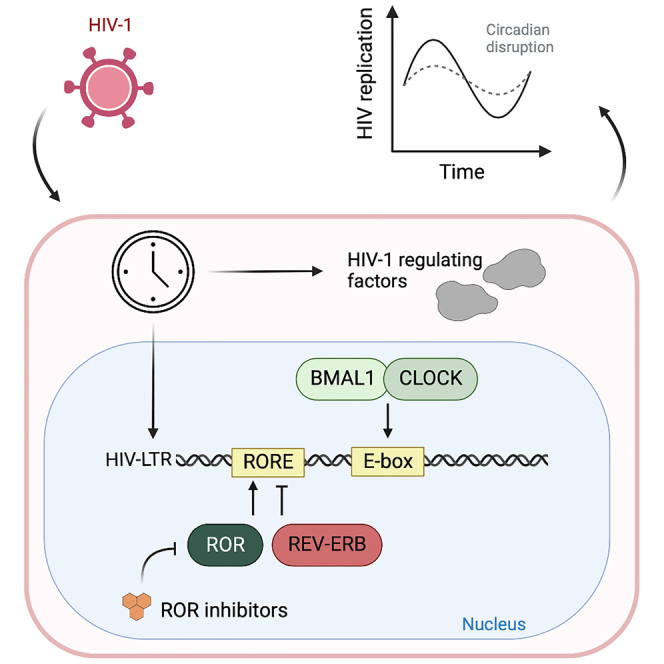

## Introduction

HIV is the causative agent of acquired immunodeficiency syndrome (AIDS), and despite the increase in access to antiviral treatments globally, there remains no cure. Integration of the viral genome into host chromatin is an essential step in the replicative life cycle, and infection is dependent on the host transcriptional machinery.^1^ Viral transcription is regulated by the 5′ long terminal repeat (LTR) region which contains regulatory elements that recruit host transcriptional activators and repressors.^2^ Current anti-retroviral therapies (ARTs) target multiple steps of the HIV-1 life cycle to suppress replication but fail to eradicate the long-lived reservoirs of viral integrants which perpetuate infection.^3^ HIV-1 infects mainly hematopoietic CD4 expressing cells that comprise the main site of virus replication, with T helper 17 (Th17) cells being particularly susceptible to infection.^4^ Th17 cell differentiation depends on the master transcriptional regulator retinoic acid related-orphan receptor C (RORC)^5^ that was recently reported to promote HIV-1 replication,^6^ highlighting the importance of understanding the cellular transcriptional landscape in defining viral tropism.

The circadian clock is an endogenous timing system that oscillates with a ∼24 h period and coordinates many physiological processes. To anticipate environmental changes and minimize the risk of infection, immune functions depend on the cellular clock with many parameters exhibiting diurnal oscillations.^7^ The outcome of infection by a wide range of pathogens is influenced by the time of day, and disruption of the circadian clock can increase disease severity.^8^ On a molecular level, each cell has its own intrinsic clock machinery, which is orchestrated by several interlinked transcriptional/translational feedback loops.^9^ The main circadian activators, brain and muscle ARNT-like 1 (BMAL1) and circadian locomotor output cycles kaput (CLOCK), form heterodimers that bind *cis* elements called E-boxes in the regulatory region of target genes which include their own repressors Period (*Per*) and Cryptochrome (*Cry*). An ancillary feedback loop is composed of the nuclear receptors REV-ERBs and RORs, themselves under transcriptional activation by CLOCK/BMAL1. REV-ERBs and RORs bind to ROR response elements (ROREs) in the promoter regions of target genes, notably *Bmal1*, to inhibit or activate transcription, respectively.^10^ Multiple small molecule modulators targeting core clock proteins (mainly CRYs, REV-ERBs, or RORs) have been discovered and have potential for therapeutic avenues.^11^ In addition, optimizing the time of day for medicine and vaccine delivery can influence treatment efficacy and disease outcomes.^12^

Viruses are reliant on their hosts for replication, and recent reports highlight the role of circadian systems in viral infection.^13^^,^^14^^,^^15^ Clinical studies show an association between HIV-1 RNA and transcript levels of BMAL1^16^ and a rhythmic pattern of peripheral HIV-1 RNA in patients on ART.^17^ These studies illustrate an association; however, it remains to be elucidated if diurnal changes in HIV RNA are a result of cell-intrinsic clock regulation. We demonstrate that HIV-1 replication is rhythmic in an *in vitro* cellular system and provide evidence of BMAL1, REV-ERBα and RORC binding to the viral genome. Pharmacological inhibition of RORC using inverse agonists perturbed the cellular clock and inhibited HIV-1 transcription. We further show that host factors important for HIV-1 replication are clock regulated, and viral replication can be perturbed by ROR modulation. Our work provides fundamental insights on the core circadian transcription factors and circadian-regulated host pathways that regulate HIV replication.

## Results

### HIV-1 replication is rhythmic

**Figure 1:**
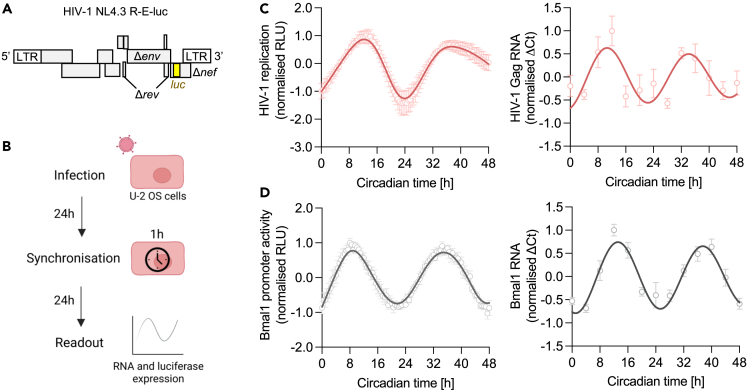
HIV-1 replication is rhythmic (A) Cartoon of the HIV-1 NL4.3 R-E-luc (NL4.3-luc) reporter, encoding HIV genes with flanking long terminal repeats (LTR), including defective envelope (*Δenv*), regulator of expression of virion proteins (*Δrev*) and negative regulator factor (*Δnef*) and the luciferase (*luc*) gene which is the readout for viral replication. (B) U-2 OS cells were infected with HIV-1 NL4.3-luc VSV-G for 24 h followed by serum shock synchronization for 1 h. 24 h later, luciferase activity was measured at 30 min intervals or cells harvested at 4 h intervals for RNA extraction for a total of 48 h. (C) U-2 OS cells were infected with HIV-1 NL4.3-luc VSV-G, synchronized and HIV-1 replication measured by luciferase activity (mean ± S.E.M., n = 6) or cells were harvested at 4 h intervals for RNA extraction and HIV-1 Gag transcripts measured relative to a B2M housekeeper by qPCR (mean ± S.E.M., n = 4). Analysis of luciferase data: eJTK cycle p < 0.00001, period = 24.7 h, peak expression = 12.2 h (FFT-NLLS analysis, BioDare2). (D) U-2 OS cells stably expressing luciferase under control of the Bmal1 promoter (Bmal1-luc) were synchronized and promoter activity measured at 30 min intervals (mean ± S.E.M., n = 7). Wild-type U-2 OS cells were synchronized, harvested at 4 h intervals, followed by RNA extraction and qPCR detection of Bmal1 RNA relative to a B2M housekeeper (mean ± S.E.M., n = 4). All data are normalized to peak expression.

To investigate a role for circadian pathways to regulate HIV-1 infection, we used the human osteosarcoma cell line U-2 OS since they show robust oscillations *in vitro* following serum shock^18^ and are widely used in circadian research.^19^ The single-cycle HIV-1 reporter NL4.3 R-E-luc (NL4.3-luc) encodes luciferase as a readout for viral replication (Figure 1A) and provides a tool to quantify HIV-1 transcription without generating progeny virus and secondary reinfection events.^20^ Complementing the glycoprotein-defective NL4.3-luc with vesicular stomatitis virus encoded G protein (VSV-G) generates pseudoparticles that can infect a broad range of cell types and bypasses the natural HIV entry receptors (CD4 and chemokine receptors).^21^ We show that U-2 OS cells support HIV-1 replication that requires integration into the host genome as evidenced by the antiviral activity of the integrase inhibitor raltegravir^22^ (Figure S1). To assess whether HIV-1 replication is rhythmic, U-2 OS cells were infected with NL4.3-luc VSV-G and synchronized by serum shock. Cellular RNA was harvested at 4 h intervals to quantify HIV-1 Gag RNA by qPCR, and luciferase activity measured at 30 min intervals (Figure 1B). We observed rhythmic HIV-1 replication over 48 h (Figure 1C) with a period of 24.7 h and peak viral replication at circadian time (CT) 12.2 h (p < 0.00001, fast Fourier transform non-linear least squares analysis FFT-NLLS^23^ in BioDare2^24^). U-2 OS cells stably expressing luciferase under control of the Bmal1 promoter (Bmal1-luc) enabled us to compare HIV and Bmal1 transcriptional activity. Bmal1 promoter activity and transcript levels displayed a similar period and peak expression to HIV-1 replication (Figure 1D). Synchronization of U-2 OS cells was confirmed by assessing transcript levels of additional clock genes (Figure S2).

To extend these observations to a more physiologically relevant cell type, we attempted to synchronize a Jurkat CD4 T cell line and human primary CD4 T cells by serum shock (Figure S3). As previously reported, these cells failed to show robust circadian changes when measuring Bmal1 transcripts, despite being rhythmic in circadian-entrained mice and individuals.^25^ In summary, we show that U-2 OS cells are a suitable *in vitro* model system to study HIV-1 infection and that viral transcription is rhythmic, highlighting a role for the host circadian machinery in regulating HIV-1.

### BMAL1 regulates rhythmic HIV replication through binding its genomic DNA

**Figure 2:**
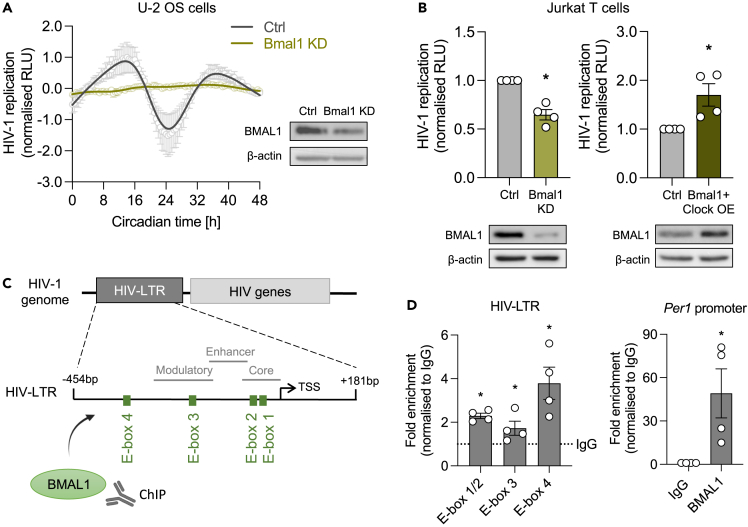
BMAL1 regulates HIV-1 replication (A) U-2 OS parental control (ctrl) cells or U-2 OS Bmal1 knockdown (KD) cells generated by shRNA-mediated silencing were infected with HIV-1 NL4.3-luc VSV-G. 24 h later, infected cells were synchronized by serum shock, and viral replication measured by luciferase readout (mean ± S.E.M., n = 3, normalized to peak, raw data in Figure S9A). BMAL1 and β-actin protein levels were assessed by western blotting (representative of n = 2). (B) Jurkat cells were infected with HIV-1 NL4.3-luc VSV-G for 24 h and Bmal1 KD or Bmal1/Clock over-expression (OE) was generated by siRNA-mediated silencing (scrambled siRNA as ctrl) or transfection of plasmids (pcDNA3.1 as ctrl). 48 h post transfection, viral replication was measured by luciferase expression (mean ± S.E.M., n = 4, Mann-Whitney test, normalized to ctrl) and BMAL1 and β-actin protein levels assessed by western blotting (representative of n = 2). (C) Cartoon of the 5′ HIV-long terminal repeat (LTR) which encodes four E-box motifs (E-box 1: CAGCTG, E-box 2: CAGATG, E-box 3: CACATG, E-box 4: CAGTTG) in the NL4.3 strain; base pairs (bp) are shown relative to the transcriptional start site (TSS). (D) Binding of BMAL1 to the HIV-LTR was assessed by chromatin immunoprecipitation (ChIP). Jurkat cells were infected with NL4.3-luc VSV-G, and chromatin extracts immunoprecipitated with BMAL1 antibody or rabbit IgG as a negative control. Fold enrichment of binding to either the E-boxes in the HIV-LTR or to the *Per1* promoter as a positive control was quantified by qPCR and is shown compared to non-specific binding of IgG (mean ± S.E.M., n = 4, Mann-Whitney test).

Bmal1 is the only core clock gene whose deletion is sufficient to cause arrhythmicity of locomotor activity—the hallmark of circadian disruption.^26^^,^^27^ We noticed a temporal synchronicity in the transcriptional profiles of Bmal1 and HIV-1, leading us to assess the role of BMAL1 in HIV-1 infection. Rhythmic HIV-1 replication was blunted in Bmal1 knockdown (KD) cells compared to the parental control (Figure 2A). Furthermore, silencing of Bmal1 or over-expression (OE) of BMAL1 and CLOCK in Jurkat cells resulted in a reduction or increase in HIV-1 replication compared to the control cells, respectively (Figure 2B). The HIV-1 LTR encodes four E-box motifs in the HIV NL4.3 strain (Figure 2C), and earlier reports showed that mutating these sites impaired HIV transcription,^16^^,^^28^ supporting a model where BMAL1 binds and activates the HIV-LTR. To test this hypothesis, we isolated chromatin from NL4.3-luc VSV-G-infected Jurkat cells and performed a BMAL1 chromatin immunoprecipitation (ChIP) assay. Primers designed to amplify specific E-boxes in the HIV-1 LTR showed enriched binding of BMAL1 to all motifs and to the *Per1* promoter (Figure 2D). Overall, these data provide evidence that BMAL1 binding to the HIV-LTR enhances HIV-1 transcription.

### ROR inhibition modulates host circadian clock factors and HIV-1 replication

**Figure 3:**
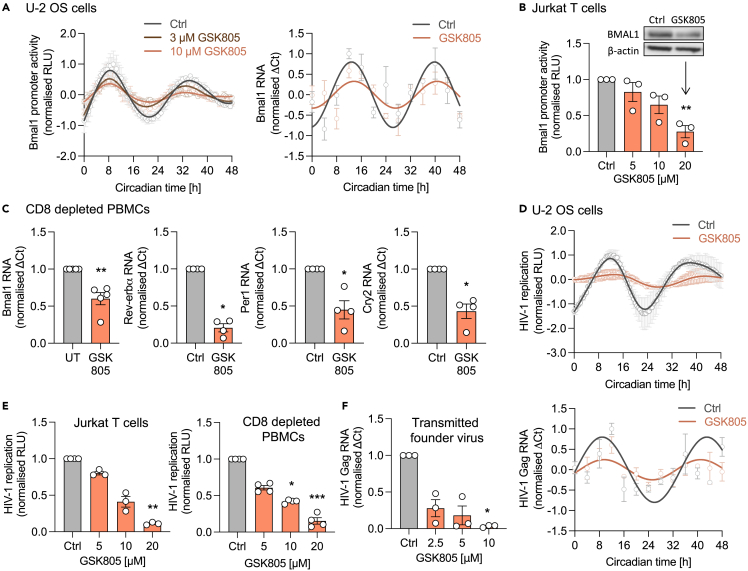
ROR inhibition modulates host circadian clock factors and HIV-1 replication (A) U-2 OS cells stably expressing luciferase under control of the Bmal1 promoter (Bmal1-luc) were synchronized followed by treatment with GSK805. Luminescence was measured at 30 min intervals (mean ± S.E.M., n = 3, raw data in Figure S9B). Amplitude reduction compared to UT: 36.1% for 3 μM, 63.9% for 10 μM (FFT-NLLS analysis, BioDare2). Synchronized parental U-2 OS cells were treated with GSK805 (10 μM) and harvested at 4 h intervals, followed by RNA extraction and qPCR detection of Bmal1 transcripts relative to a B2M housekeeper (mean ± S.E.M., n = 3). (B) Jurkat cells stably expressing Bmal1-luc were treated with GSK805 for 24 h, and luciferase activity quantified (mean ± S.E.M., n = 3, Kruskal-Wallis ANOVA). Jurkat cells were treated with GSK805 (20 μM), with BMAL1 and β-actin protein expression assessed by western blotting (representative of n = 3). (C) CD8-depleted PBMCs were activated for 3 days with anti-CD3/CD28, treated with GSK805 (2.5 μM) for 7 days, followed by RNA extraction, and qPCR detection of Bmal1, Rev-erbα, Per1, or Cry2 RNA levels relative to a B2M housekeeper (mean ± S.E.M., n = 4–5, Mann-Whitney test). (D) U-2 OS cells infected with NL4.3-luc VSV-G were synchronized, treated with GSK805 (10 μM) and luciferase activity measured at 30 min intervals (mean ± S.E.M., n = 3, normalized to peak) or cells harvested at 4 h intervals, followed by qPCR detection of HIV-1 Gag RNA relative to a B2M housekeeper (mean ± S.E.M., n = 3, raw data in Figure S9C). (E) Jurkat cells or activated CD8-depleted PBMCs were infected with NL4.3-luc VSV-G, treated with GSK805 for 24 h and luciferase quantified as a readout of HIV-1 replication (mean ± S.E.M, n = 3–4, Kruskal-Wallis ANOVA). (F) Activated CD8-depleted PBMCs were spinoculated for 2 h with patient-derived HIV-1 (transmitted founder virus clone CH185) and cultured with GSK805 for 7 days, followed by qPCR detection of HIV-1 Gag RNA relative to B2M housekeeper (mean ± S.E.M., n = 3, Kruskal-Wallis ANOVA). All data are expressed relative to UT control.

Pharmacological agents targeting core circadian factors provide tools to study the mechanism(s) underlying the circadian regulation of HIV-1. We previously showed that REV-ERB agonists inhibit HIV-1 replication whilst antagonism increased LTR activity.^28^ While compounds directly targeting BMAL1 are not currently available, several drugs targeting RORs, regulators of *Bmal1* expression, have been developed.^11^ Interestingly, the RORC isoform regulates Th17 cell differentiation,^5^ and Wiche Salinas et al. recently reported that the RORC inverse agonist GSK805 inhibits HIV-1 replication.^6^ However, our knowledge of how ROR inverse agonists impact the cellular clock or perturb rhythmic HIV replication is limited.^11^^,^^29^ Both U-2 OS and Jurkat cells express RORC (Figure S4A). Treating synchronized U-2 OS cells with GSK805 showed a dose-dependent dampening of rhythmic Bmal1 promoter activity and endogenous transcript levels, with a 63.9% reduction in their amplitude at 10 μM compared to untreated cells (Figure 3A). We also noted a reduction in the rhythmic expression of several key circadian genes (*Rev-erbα, Per1, and Cry2*), all of which encode RORC binding motifs in their promoter regions (Figures S4B and S4C). Reassuringly, GSK805 treatment showed a dose-dependent inhibition of Bmal1 promoter activity and protein expression in Jurkat cells (Figure 3B). To extend our observations to physiologically relevant cell types, we treated CD8-depleted human peripheral blood mononuclear cells (PBMCs) with GSK805 and showed a reduction in *Bmal1, Rev-erbα, Per1,* and *Cry2* transcripts (Figure 3C), showing a general perturbation of circadian gene expression.

**Figure 4:**
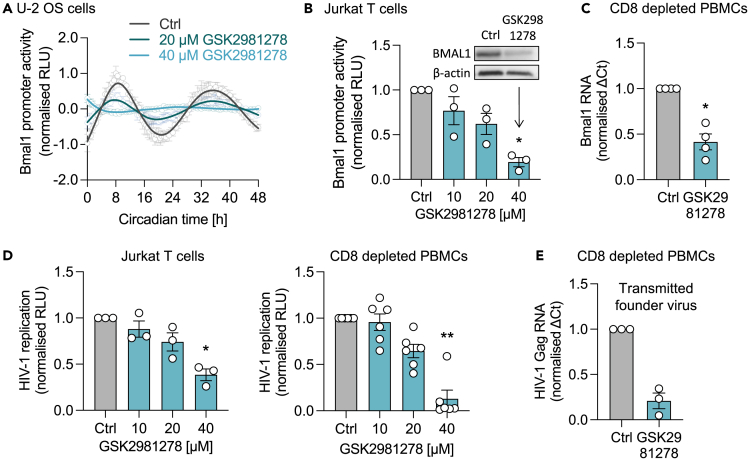
An independent ROR inhibitor reduces Bmal1 expression and HIV-1 replication (A) U-2 OS cells stably expressing luciferase under control of the Bmal1 promoter (Bmal1-luc) were synchronized, treated with GSK2981278 and luciferase activity measured at 30 min intervals (mean ± S.E.M., n = 4, raw data in Figure S9D). Amplitude reduction compared to UT: 63.9% for 20 μM, 88.9% for 40 μM (FFT-NLLS analysis, BioDare2). (B) Jurkat cells stably expressing Bmal1-luc were treated with indicated concentrations of GSK2981278 for 24 h and luciferase activity quantified (mean ± S.E.M., n = 3, Kruskal-Wallis ANOVA). Wild-type Jurkat cells were treated with GSK2981278 (40 μM), and BMAL1 and β-actin protein expression assessed by western blotting (representative of n = 3). (C) CD8-depleted PBMCs were activated for 3 days with anti-CD3/CD28, cultured in the presence of GSK2981278 (20 μM) for 7 days, followed by RNA extraction and qPCR detection of Bmal1 RNA relative to a B2M housekeeper (mean ± S.E.M., n = 4, Mann-Whitney test). (D) Jurkat cells (mean ± S.E.M., n = 3, Kruskal-Wallis ANOVA) or activated CD8-depleted PBMCs (mean ± S.E.M., n = 6, Kruskal-Wallis ANOVA) were infected with NL4.3-luc VSV-G, treated with different doses of GSK2981278 for 24 h and luciferase quantified as readout of HIV-1 replication. (E) Activated CD8-depleted PBMCs were spinoculated for 2 h with patient-derived HIV-1 (transmitted founder virus clone CH185), cultured in medium with GSK2981278 (20 μM) for 7 days, followed by RNA extraction and qPCR detection of HIV-1 Gag RNA relative to a B2M housekeeper (mean ± S.E.M., n = 3). All data are expressed relative to UT control.

As GSK805 disrupts the cellular circadian system, we hypothesized that this compound would inhibit rhythmic viral replication. GSK805 treatment dampened rhythmic HIV replication and viral RNA in U-2 OS cells (Figure 3D). GSK805 also inhibited HIV replication in Jurkat and CD8-depleted PBMCs (Figure 3E). To extend these observations we used a patient-derived founder strain of HIV-1 (CH185) (Figure S3D), and GSK805 treatment reduced the levels of viral transcripts (Figure 3F). Of note, the compound showed no cytotoxicity at the concentrations tested in all cell types (Figure S5). To extend and validate these studies we showed that a chemically distinct RORC inhibitor, GSK2981278,^30^ inhibited rhythmic Bmal1 promoter activity in U-2 OS and reduced the amplitude by 88.9% (40 μM) (Figure 4A). We also observed a dose-dependent reduction in Bmal1 promoter activity and protein expression in Jurkat cells (Figure 4B) and reduced Bmal1 transcript levels in CD8-depleted PBMCs (Figure 4C), with no evidence for cytotoxicity (Figure S6). GSK2981278 inhibited the replication of HIV strains NL4.3 and CH185 in Jurkat and primary cells (Figures 4D and 4E). Collectively, these data show that pharmacologically targeting RORC disrupts the cellular clock and inhibits rhythmic HIV-1 replication.

### Differential binding of REV-ERBα and RORC to the HIV-LTR at different circadian times

**Figure 5:**
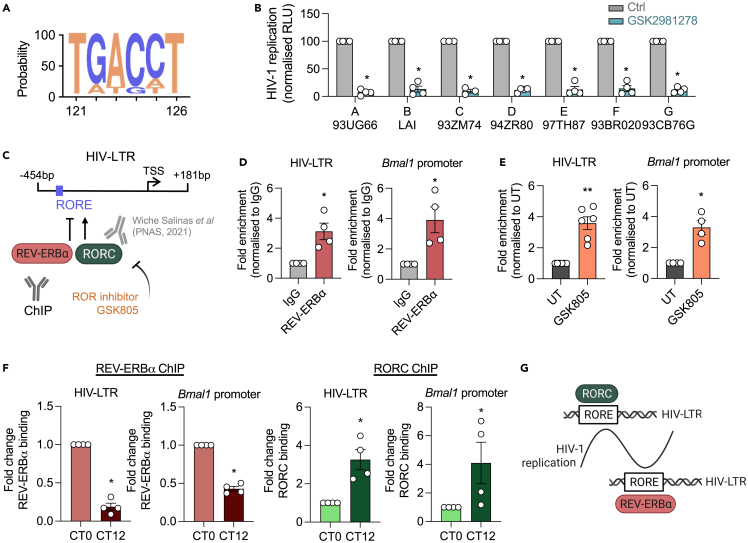
Differential binding of REV-ERBα and RORC to the HIV-LTR at different circadian times (A) Consensus plot showing the conservation of nucleotides in position 121 to 126 of 1266 HIV-1 sequences deposited in the Los Alamos Database (coordinates are from the HXB2 reference). Bases of coding strand are displayed which correspond to the nucleotides ‘AGGTCA’ on the antisense strand, thereby representing the ROR response element (RORE) ‘RGGTCA’. Conservation is reflected by the height of the bases (y axis 0–100%). (B) Jurkat cells were transfected with reporter constructs of HIV-1 subtypes A-G, followed by GSK2981278 treatment (40 μM) and LTR activity measured by quantifying luciferase activity 24h later (mean ± S.E.M., n = 4, Mann-Whitney test). (C) Location of the RORE in the 5′ HIV-LTR of NL4.3 strain; base pairs (bp) are shown relative to the transcriptional start site (TSS). Model where ROR and REV-ERBα compete for binding to the HIV-LTR which can be tested by chromatin immunoprecipitation (ChIP). RORC binding to the HIV-LTR was shown by Wiche Salinas et al.^6^ (D) Jurkat cells were infected with NL4.3-luc VSV-G for 24 h, and chromatin extracts immunoprecipitated with anti-REV-ERBα or rabbit IgG as a negative control. Fold enrichment of binding to the RORE in either the HIV-LTR or the Bmal1 promoter was quantified by qPCR and is shown compared to the IgG control (mean ± S.E.M., n = 4, Mann-Whitney test). (E) Jurkat cells were infected with NL4.3-luc VSV-G followed by treatment with GSK805 (10 μM) for 24 h. Fold enrichment of REV-ERBα binding to the HIV-LTR RORE or the Bmal1 promoter was compared between untreated and GSK805-treated cells (mean ± S.E.M., n = 4–6, Mann-Whitney test). (F) U-2 OS cells were infected with HIV-1 NL4.3-luc VSV-G for 24 h followed by serum shock synchronization for 1 h. 24 h (CT0), and 36 h (CT12) post-synchronization cells were harvested, and chromatin extracts immunoprecipitated with REV-ERBα antibody, RORC antibody, or rabbit IgG as a negative control. Fold enrichment of binding to either the RORE in the HIV-LTR or to the Bmal1 promoter as a positive control was quantified by qPCR, normalized to non-specific IgG binding and shown as fold change of binding (mean ± S.E.M., n = 4, Mann-Whitney test). (G) Model where RORC binding to the HIV-LTR promotes HIV-1 replication, and REV-ERBα binding supresses viral replication at different circadian times resulting in rhythmic HIV-1 replication. See related Figure S7. All data are expressed relative to the control cells.

RORC has been shown to regulate HIV replication through direct binding to an RORE in the HIV-LTR.^6^ The RORE motif is highly conserved among diverse LTR sequences (Figure 5A), and screening a panel of HIV-LTR-luc reporter plasmids, representing the major HIV-1 clades A-G strains,^28^ showed similar sensitivity to ROR inverse agonist treatment (Figure 5B). Since ROR can compete with the circadian repressor REV-ERB for binding to ROREs^31^ and silencing of REV-ERBs enhanced HIV-LTR activity,^28^ we hypothesized that REV-ERB binds to the HIV-LTR. To evaluate this, we isolated chromatin from HIV-1-infected Jurkat cells, and ChIP analysis showed REV-ERBα binding to the HIV-LTR and its host target Bmal1 promoter (Figures 5C and 5D). ROR inhibition by the inverse agonist GSK805 increased REV-ERBα binding to the HIV-LTR (Figure 5E), supporting our model that RORC and REV-ERBα compete to regulate HIV transcription through the RORE element. To elucidate the dynamics of REV-ERBα and RORC binding, we harvested chromatin from synchronized infected U-2 OS cells at CT0 or CT12. The binding of REV-ERBα to the HIV-LTR and endogenous Bmal1 promoter was higher at CT0 than CT12, whereas RORC showed the opposite phenotype (Figures 5F and S7). These data are consistent with the pattern of rhythmic HIV-1 replication showing a trough and peak at CT0 and CT12, respectively. We propose a model where binding of the activator RORC promotes HIV-1 replication, while REV-ERBα reduces viral replication at alternate circadian times, which results in rhythmic HIV-1 replication (Figure 5G). Overall, we demonstrate a temporal interplay between REV-ERBα and RORC binding to the HIV-LTR to regulate rhythmic HIV-1 replication.

### HIV-1 host factors are circadian regulated

**Figure 6:**
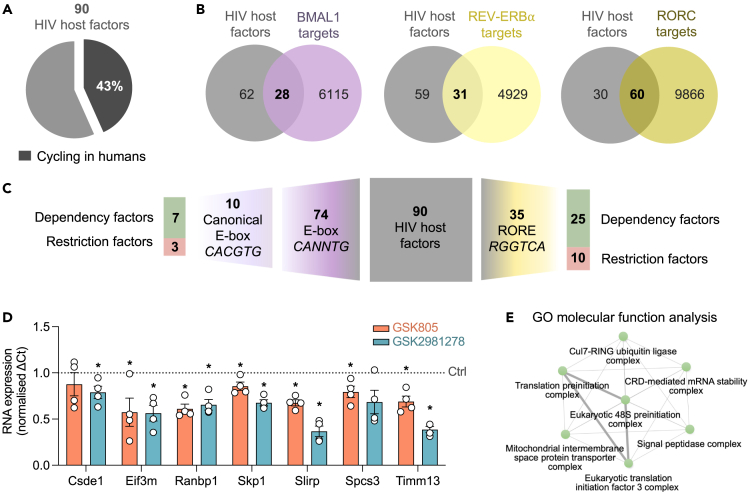
HIV-1 host factors are regulated by the circadian clock (A) Expression of 90 HIV-1 host proteins^34^ was analyzed using the Circa database,^35^ and 43% of genes identified as cycling in humans. (B) BMAL1-regulated genes,^36^ REV-ERBα-regulated genes,^37^ and RORC-regulated genes^38^ were compared with host factors known to alter HIV-1 replication. (C) HOMER (Hypergeometric Optimization of Motif EnRichment tool^39^) was used to analyze -1kb promoter regions of HIV-1 host factors and identified gene promoters encoding E-box motifs or ROR response elements (ROREs). (D) Jurkat cells were treated with GSK2981278 (40 μM) or GSK805 (20 μM) for 24 h, cells were lysed, RNA was extracted, and gene expression analyzed via qPCR (mean ± S.E.M., n = 4, Mann-Whitney test). (E) Gene ontology (GO) cellular components analysis^40^ where each node represents an enriched GO term. Related GO terms are connected by lines, where thickness reflects the percentage of overlapping genes. See related Figure S8.

HIV-1 replication is dependent on a myriad of host factors, many of which could be affected by circadian rhythms. Several studies have identified cellular proteins that positively (dependency factors) or negatively (restriction factors) influence HIV replication.^32^^,^^33^ Using a high-throughput CRISPR-Cas9 gene-editing approach, Hiatt et al. identified 90 genes that regulate HIV replication in human CD4 T cells.^34^ To assess whether the expression of these host factors is rhythmic, we interrogated the Circa database^35^ and showed that 43% of genes are cycling in multiple human tissues (Figures 6A and S8A). To extend our analysis, we examined several experimentally validated ChIP sequencing (ChIP-seq) datasets: including BMAL1-regulated genes from the mouse liver^36^ and REV-ERBα^37^- or RORC^38^-regulated genes from murine Th17 cells. Comparing the BMAL1-, REV-ERBα-, and RORC-regulated target genes with the 90 HIV host factors^34^ showed 28 BMAL1-, 31 REV-ERBα-, and 60 RORC-regulated HIV host factors (Figures 6B, S8B, and S8C). To identify HIV-1-associated host factors which are likely to be under direct BMAL1, REV-ERB, or ROR control, we analyzed their promoter regions for the presence of circadian motifs using HOMER (Hypergeometric Optimization of Motif EnRichment tool^39^). We identified 35 genes encoding an RORE and 74 genes with an E-box (CANNTG), with 10 having the canonical E-box motif (CACGTG, Figure 6C). Notably, 5 promoters encoded both RORE and E-box motifs (Figure S8D) giving a total of 40 genes. To validate this bioinformatic analysis, we screened all 40 genes for their response to RORC inverse agonists treatment in Jurkat cells. Seven genes were identified where GSK805 and GSK2981278 significantly reduced their expression (Figure 6D), consistent with their antiviral activity. Gene ontology (GO) cellular components analysis revealed that these 7 genes are involved in multiple cellular processes, ranging from transcriptional control to protein transport (Figure 6E).^40^ In summary, we have identified circadian-regulated host factors which potentially contribute to rhythmic HIV-1 replication and can be modulated by ROR inhibition.

## Discussion

In this study we show that HIV-1 replication is rhythmic in circadian-synchronized cells and identify a role for BMAL1 in activating viral replication via binding to the LTR. Pharmacological perturbation of circadian cycling by ROR inhibition dampened rhythmic HIV-1 replication and inhibited the transcriptional activity of LTR sequences from diverse clades, suggesting that targeting host circadian components may be a pan-genotypic antiviral strategy. Furthermore, we provide evidence that REV-ERBα binds to a conserved RORE in the viral regulatory region, and this interaction was enhanced by ROR inhibition, suggesting competition between these nuclear receptors for binding the HIV-LTR, similar to their competition for *Bmal1* and other clock-controlled genes.^31^ Binding of the repressor REV-ERBα to the HIV-LTR was higher at CT0 compared to CT12, while RORC binding showed the opposite effect. These data highlight a role for circadian pathways to regulate the dynamics of nuclear receptor binding to the HIV-1 genome and influencing rhythmic viral replication. Since the HIV-LTR encodes binding sites for both BMAL1 and REV-ERB/ROR along with a plethora of other transcription factors, the time-setting of HIV-1 replication is likely to be complex. Finally, we identify and validate circadian-regulated HIV-1 host factors which may contribute to rhythmic HIV-1 replication.

Since HIV-1 does not replicate in conventional rodent models, we used human cell culture systems to study how the cellular intrinsic clock regulates HIV replication. We were unable to synchronize CD8-depleted PBMCs or Jurkat T cells and selected U-2 OS cells as a well-characterized circadian model^19^ that were previously reported to support HIV-1 replication.^41^ As CD4 hematopoietic cells are difficult to genetically manipulate, other cell types such as HeLa^42^^,^^43^ or HEK293T^16^ have been widely used to study HIV molecular biology.

We observed that BMAL1/CLOCK OE increased HIV-1 replication in T cells, supporting a positive role for BMAL1 in driving HIV-1 transcription, consistent with an earlier report showing increased HIV promoter activity with BMAL1/CLOCK OE in HEK293T cells.^16^ We and others have shown that mutation of the E-box motifs in the LTR reduced transcriptional activity,^16^^,^^28^ suggesting a role for binding host factors that drive HIV-1 replication. Here, we show evidence of BMAL1 binding to all four E-box motifs in the LTR. However, direct mapping should be interpreted with some care as the sonication protocol used in the ChIP assay can shear chromosomal DNA into ∼200-300bp fragments.^44^ We previously reported that BMAL1 can bind E-box motifs in the hepatitis B viral genome,^15^ supporting a model where human viruses have evolved to act in concert with host circadian transcription factors. In contrast, herpes and influenza A virus infections were enhanced in BMAL1-deficient mice.^14^ These differences could result from the differential circadian modulation of viral replication in host cells vs. the antiviral immune responses in the whole animal.

*Rorc* knockout in mice was shown to decrease the amplitude of circadian oscillations in target genes without changing the phase or period.^45^ ROR inverse agonists reduced clock gene expression in U-2 OS cells and T cells, in line with previous reports on other ROR inhibitors.^29^ Importantly, we found that ROR inverse agonists reduced HIV replication and repressed the activity of LTRs from different HIV-1 clades. ROR inhibitors have been evaluated in a variety of disease and clinical models.^11^^,^^46^ For instance, GSK805 was reported to reduce encephalomyelitis symptoms in a murine model of multiple sclerosis^47^ and limit intestinal inflammation.^48^ GSK2981278 was evaluated in human clinical trials for the treatment of psoriasis.^30^^,^^46^ These studies indicate clinical potential, but therapeutic exploitation of circadian compounds to modulate HIV-1 infection and their potential systemic effects on the host circadian rhythm requires further evaluation.

HIV-1 replication depends on a range of cellular host factors,^32^^,^^33^ and we propose that many of these are circadian regulated. Cleret-Buhot et al. analyzed the expression of HIV-1 dependency factors between Th1 and Th17 T cells and observed increased *Rorc* and *Bmal1* transcripts in Th17 cells and an enrichment of REV-ERBα canonical pathways compared with Th1 cells.^49^ An additional study by Hiatt et al.^34^ performed CRISPR-Cas9 silencing of previously identified HIV-host protein-protein interactions in CD4 T cells, which served as the basis for our analysis. We found that nearly half of the HIV-1 host factors are cycling in humans; however, this does not account for tissue-specific gene expression. Comparing the list of HIV-1 host factors with validated BMAL1, REV-ERBα, and RORC target genes in murine liver or T cells identified several potential circadian-regulated HIV-1 host factors. Given species and tissue-specific differences in circadian gene expression,^50^^,^^51^ these datasets can be further improved and reflect the limited circadian studies in human immune cells.

Bioinformatic analysis identified E-box or RORE motifs in the promoter regions of many known HIV-1 host factors, consistent with the circadian regulation of these genes. However, a more distant circadian regulation in the context of 3D chromatin structure is possible.^50^ We validated the effect of both ROR inhibitors on these genes and identified seven hits: *Csde1, Eif3m, Ranbp1, Skp1, Slirp, Spcs3,* and *Timm13*. All of these factors were previously identified based on their interaction with viral proteins,^33^^,^^34^ and there is further evidence for their role in HIV-1 replication or circadian rhythms. The RNA-binding protein CSDE1 co-localized with HIV-1 RNA in infected HeLa cells,^52^ and the eukaryotic translation initiation factor 3 (EIF3) has been shown to limit HIV-1 replication.^33^^,^^43^ RAN binding protein 1 (RANBP1) is important for posttranscriptional regulation of HIV-1,^53^ and its silencing inhibits HIV-1 replication,^42^ which is in line with our observations. As part of a ubiquitin ligase complex involved in the degradation of CRYs, S-phase kinase associated protein 1 (SKP1) impacts cellular rhythmicity.^54^ There is limited evidence for signal peptidase complex subunit 3 (SPCS3) in regulating the cellular clock; however, it has been reported to play a role in flavivirus genesis.^55^ Overall, these circadian-regulated host factors provide a basis for future analysis to elucidate mechanisms defining rhythmic HIV-1 replication.

Our study focused on the influence of clock factors on HIV transcription; however, other stages of the viral life cycle may also be influenced by circadian pathways. For example, one of the HIV-1 entry receptors C-X-C chemokine receptor 4 (CXCR4) showed diurnal variation^56^ which could result in time-of-day-dependent viral entry. We primarily investigated the influence of BMAL1, REV-ERB, and ROR on HIV-1 replication; however, other circadian factors could also play a role as a Per1 short isoform was shown to inhibit HIV-1 transcription in resting CD4 T cells.^57^

Can HIV infection perturb the host circadian clock? The HIV-encoded *trans*-activator of transcription (Tat) protein can modulate circadian rhythmicity;^58^^,^^59^ however, the mechanism was not defined. In contrast, Stern et al. showed intact circadian transcriptional machinery in T cells from HIV-infected subjects.^17^ Clinical studies report an attenuation of diurnal blood pressure rhythms in HIV-infected patients that may be due to their compromised immune status.^60^^,^^61^ Premature immune aging and non-AIDS-related diseases increase in HIV patients with age.^62^ Importantly, rhythmic activities such as sleep/wake patterns change with increasing age,^63^ consistent with reports of disturbed sleep in HIV infection^64^^,^^65^ and delayed circadian rhythms in older Africans living with HIV.^66^ Our study shows a direct circadian regulation of HIV replication and highlights the complex interplay among the clock, aging, and sleep in HIV therapy.

HIV-1 establishes chronic life-long infection, and it is beneficial for the virus to adapt to the host’s physiology, including its endogenous circadian rhythms. This is reflected by the balance between the host immune response and viral evasion strategies to reduce immune-associated costs. For instance, inflammatory responses in humans are reduced during the night (resting phase) when low pathogen encounter is anticipated.^7^ We speculate that adaptation to host circadian-regulated pathways is advantageous for HIV as it maximizes viral replication during times when the host antiviral state is low. Our data show that HIV-1 replication coincides with peak Bmal1 expression that aligns with a clinical study reporting high Bmal1 and HIV-1 transcript levels in CD4 T cells collected at night,^17^ supporting viral adaptation to the host clock. However the impact of rhythmic antiviral immune response is not well understood.

Overall, our findings highlight the complex virus-circadian interplay and provide fundamental insights into the host pathways that regulate HIV-1 transcription. In-depth studies of circadian modifiers with antiviral properties could uncover novel drug targets which may augment existing treatment regimens for HIV and other viral infections. We hypothesize that viruses have evolved to adopt and exploit the cellular circadian machinery which strengthens the importance of future work to understand the viral-circadian interplay that will inform the design and evaluation of new therapies.

### Limitations of the study

One limitation of our study is the use of U-2 OS osteosarcoma cancer-derived cells that do not reflect the natural reservoir of immune cells that HIV would infect *in vivo*. Secondly, our ChIP protocol does not allow direct mapping of transcription factors binding to the HIV-LTR due to the length of fragments generated. Finally, some of our bioinformatic analyses used murine datasets that may differ from human cell lineages.

## STAR★Methods

### Key resources table

**Table:** 

REAGENT or RESOURCE	SOURCE	IDENTIFIER
**Antibodies**		

Rabbit anti-BMAL1 (WB)	Abcam	Cat# Ab93806; RRID: AB_10675117
Mouse anti-β-actin (WB)	Sigma	Cat# A5441; RRID: AB_476744
Rat anti-RORC (WB and ChIP)	eBioscience	AFKJS-9
Rabbit anti-BMAL1 (ChIP)	Abcam	Cat# Ab3350, RRID: AB_303729
Rabbit anti-NR1D1 (ChIP)	Proteintech	14506-I-AP
Rabbit IgG (ChIP)	Sigma	NI01

**Bacterial and virus strains**		

NL4.3-R-E-luc	NIBSC AIDS Repository	ARP2128
CH185 transmitted founder virus	Kind gift from Dr John Kappes	CH185

**Biological samples**		

Peripheral blood mononuclear cells	NHS Blood and Transplant, Oxford	PBMC

**Chemicals, peptides, and recombinant proteins**		

Raltegravir	Cambridge Bioscience Ltd	HY-10353
GSK805	Cambridge Bioscience Ltd	9002444
GSK2981278	Cambridge Bioscience Ltd	20974
VivoGlo Luciferin	Promega	P1041
SuperSignal West Dura Extended Duration Substrate	Thermo Fisher	34076
Live Dead Fixible Aqua stain	Life technologies	50-112-1526

**Critical commercial assays**		

CytoTox 96 Non-Radioactive Cytotoxicity Assay	Promega	G1780
cDNA Synthesis Kit	PCR Biosystems Ltd	PB30.11-10
qPCRBIO SyGreen Blue Mix	PCR Biosystems Ltd	PB20.17-20
Firefly Luciferase Assay kit	Promega	E1501

**Experimental models: Cell lines**		

HEK293T	ATCC	CRL-3216
U-2 OS	ATCC	HTB-96
Jurkat	Kind gift from Professor Xiaoning Xu	N/A

**Oligonucleotides**		

Primers	See Table S1	Life Technologies

**Recombinant DNA**		

pABpuro-BluF	Addgene	46824
pMD2G	Addgene	12259
psPAX2	Addgene	12260
pcDNA3.1	Thermo Fisher	V79020
HIV-LTR subtype luciferase constructs	Kind gift from Professor Bill Paxton	N/A
Bmal1 expression plasmid	Kind gift from Professor Ximing Qin	N/A
Clock expression plasmid	Kind gift from Professor Ximing Qin	N/A

**Software and algorithms**		

Prism 9	GraphPad	https://www.graphpad.com
eJTK_cycle	BioDare2	https://biodare2.ed.ac.uk
Fast Fourier Transform Non-linear Least Squares	BioDare2	https://biodare2.ed.ac.uk
HOMER	Hypergeometric Optimization of Motif EnRichment	http://homer.ucsd.edu/homer/

### Resource availability

#### Lead contact

Further information and requests for resources and reagents should be directed to and will be fulfilled by the lead contact Jane A McKeating (jane.mckeating@ndm.ox.ac.uk).

#### Materials availability

This study did not generate new unique reagents.

### Experimental model and subject details

#### Cell lines and primary cells

HEK293T cells and human bone osteosarcoma epithelial cells (U-2 OS) were purchased from ATCC and cultured in Gibco DMEM medium (high glucose, GlutaMAX supplement, pyruvate; Life Technologies) containing 10% FBS and 1% penicillin/streptomycin (Life Technologies). U-2 OS cells stably expressing the Bmal1 promoter upstream of luciferase (Bmal1-luc) were generated using the pABpuro-BlucF plasmid (Addgene #46824) and maintained in 2 μg/ml puromycin (Life Technologies). Jurkat T cells were provided by Professor Xiaoning Xu (Imperial College, London, UK) and maintained in RPMI-1640 medium (Life Technologies) containing 10% FBS and 1% penicillin/streptomycin (Life Technologies).

Peripheral blood mononuclear cells (PBMCs) were isolated from leukapheresis cones purchased from NHS Blood and Transplant (Oxford, UK), with written informed consent from all donors and ethical approval for research use. CD8 T cells were depleted using a kit from Miltenyi Biotec (CD8 MicroBeads, human) and cells cultured in RPMI-1640 (Life Technologies) containing 10% FBS, 1% penicillin/streptomycin (Life Technologies), 1% sodium pyruvate (Sigma), 1% Glutamax (Life Technologies), 1% non-essential aminoacids (Life Technologies) and 2 mM beta-mercaptoethanol (Life Technologies). Cells were stimulated with 50 IU/ml IL-2 (Proleukin; Novartis), 0.01 μg/ml soluble human anti-CD3 (R&D; clone UCHT1) and 0.1 μg/ml soluble human anti-CD28 (Life Technologies; clone CD28.2) for 3 days before infection. All work with primary human cells was compliant with institutional guidelines.

### Method details

#### Reagents

RORC inverse agonist GSK805 (Cambridge Bioscience Ltd) and ROR inverse agonist GSK2981278 (Cambridge Bioscience Ltd) were dissolved in dimethyl sulfoxide (Life Technologies) and their cytotoxicity assessed using a lactate dehydrogenase (LDH) assay (Promega). The integrase inhibitor raltegravir was purchased from Cambridge Bioscience Ltd. Silencing RNAs for Bmal1 (ARNTL silencer select) and siRNA controls were obtained from Thermo Fisher. LIVE/Dead™ Fixable Aqua for flow-cytometry was ordered from Life Technologies.

#### Plasmids

The lentiviral packaging plasmids pMD2G (#12259) and psPAX2 (#12260) were obtained from Addgene, pcDNA3.1 from Thermo Fisher and lenti-shBmal1 plasmid from ABM. The plasmid encoding NL4.3 R-E-luc was supplied by the NIBSC AIDS Repository and the VSV-G expression plasmid as previously reported.^67^ HIV-LTR luciferase constructs encoding LTR regions cloned from diverse HIV-1 clades were a gift from Professor B. Paxton (University of Liverpool, UK) and were previously described.^28^ The Bmal1 promoter luciferase reporter vector was purchased from Addgene (#46824). The *Bmal1* and *Clock* expression plasmids were a gift from Professor Ximing Qin (Anhui University, Hefei, China).

#### Generation of viral stocks

HEK293T cells were transfected with plasmids using polyethylenimine (PEI, Polysciences). Medium was replaced 4 h post transfection with DMEM medium without antibiotics supplemented with 10 mM HEPES and virus harvested 48 h later. For NL4.3-luc VSV-G pseudovirus production, the NL4.3 R-E-luc and VSV-G plasmids were transfected together. To produce HIV-1 CH185 transmitted founder stocks, full-length infectious molecular clone plasmid DNA (originally obtained from Dr John Kappes, University of Alabama at Birmingham, USA^68^) was transfected using Lipofectamine (Life Technologies) or Fugene 6 (Promega) and supernatants harvested 3 days later. The viral stocks were quantified by measuring the reverse transcriptase (RT) activity using either a qPCR-based product-enhanced RT (PERT) assay^69^ or a colorimetric RT assay (Roche Life Sciences).

#### *In vitro* HIV-1 infection experiments

To study NL4.3-luc VSV-G replication, U-2 OS, Jurkat cells or activated CD8 depleted PBMCs were infected with NL4.3-luc VSV-G (100 U RT/ 10^6^ cells) for 24 h. Cells were washed and incubated with medium containing drugs at indicated concentrations for 24 h, followed by lysis for qPCR analysis or detection of luminescence using a Firefly Luciferase Assay kit (Promega) and a Glomax luminometer (Promega). To study authentic HIV-1 replication, an infectious molecular clone (transmitted founder virus) from patient CH185 was used. Activated CD8 depleted PBMCs were spinoculated (0.25 ng RT / 10^6^ cells) for 2 h and cultured in media with GSK805 for 7 days, followed by lysis and Gag RNA detection via qPCR.

#### RT-qPCR

Cells were lysed and RNA extracted using a RNeasy kit (QIAGEN). For experiments with infected or plasmid transfected cells, residual DNA was digested using the TURBO DNase free kit (Thermo Fisher). Equal amounts of RNA were used for cDNA synthesis (cDNA Synthesis Kit, PCR Biosystems) and qPCR performed using Fast SYBR Master Mix (PCR Biosystems) in a LightCycler96 (Roche) with the primers listed above. mRNA expression was calculated relative to Beta-2-Microglobulin (Β2M) expression using the ΔCt method.

#### Time course experiments

For experiments measuring luciferase expression in real time, U-2 OS cells were infected with VSV-G-pseudotyped HIV-1 NL4.3-luc (100 U RT/ 10^6^ cells) for 24 h or cells stably expressing a Bmal1 promoter luciferase reporter (Bmal1-luc). Cells were synchronised by serum shock with 50% FBS for 1 h, and 24 h post serum shock media was changed to DMEM lacking Phenol Red (Life Technologies), supplemented with 100 μM luciferin (VivoGlo, Promega) and each drug, respectively. Luciferase activity was measured at 30 min intervals for a period of 48 h using a CLARIOstar luminometor (BMG Labtech), with the cells kept at 37°C and 5% CO_2_. To detect rhythmic transcript levels, U-2 OS cells, Jurkat cells or activated CD8 depleted PBMCs were infected with VSV-G-pseudotyped HIV-1 NL4.3-luc (100 U RT/ 10^6^ cells) for 24 h (with non-infected cells as control), synchronised via serum shock with 50% FBS for 1 h and from 24 h later cells harvested at 4 h intervals, followed by RNA extraction and detection by qPCR as described above. Cycling datasets were analysed with BioDare2^24^ using empirical JTK_cycle (eJTK cycle^70^) and Fast Fourier Transform Non-linear Least Squares (FFT-NLLS^23^) analysis to estimate the period, phase and amplitude of cycling transcripts. All data was normalised to control peak expression and curves fitted using Prism 9 (GraphPad). It is important to note that this normalisation sets the baseline of all data to 0. Representative raw data for each experiment can be found in Figure S9.

#### Overexpression and silencing

*Bmal1* and *Clock* expression plasmids, or a pcDNA3.1 control, were delivered into Jurkat cells via transfection (ViaFect, Promega). For siRNA-mediated silencing, Bmal1 siRNA or a scrambled control were transfected using Fugene SI (Promega). 48 h post transfection cells were harvested for western blotting or luminescence detection using a Firefly Luciferase Assay kit (Promega) and a Glomax luminometer (Promega). To generate stable Bmal1 knock-down cells, U-2 OS cells were transduced with lentiviral vector encoding shBmal1 and cells selected using 2 μg/ml of puromycin (Life Technologies).

#### Western blotting

Cells were lysed using RIPA buffer (20 mM Tris, pH 7.5, 2 mM EDTA, 150 mM NaCl, 1% NP40, and 1% sodium deoxycholate), supplemented with a protease inhibitor cocktail (Roche Complete), Laemmli sample buffer was added and samples incubated at 95°C for 5 min. Proteins were separated on a 10% polyacrylamide gel and transferred to polyvinylidene difluoride membrane (Amershan Hybond P PVDF membrane, Merck). Membranes were blocked in 5% milk in PBS/0.1% Tween-20, followed by incubation with anti-BMAL1 (Ab93806, abcam), anti-RORC (AFKJS-9, eBioscience) or anti-β-actin (A5441, Sigma) primary antibodies and appropriate HRP-conjugated secondary antibodies (DAKO). A chemiluminescence substrate (West Dura, 34076, Thermo Fisher) was used to visualise proteins using a ChemiDoc XRS+ imaging system (BioRad).

#### HIV-LTR RORE and subtype LTR activity analysis

The HIV-1 5’LTR sequences deposited in the Los Alamos Database were searched in the region corresponding to the RORE (HXB2 residues 121 – 126). The program AnalyzeAlign (www.hiv.lanl.gov) was used to analyse 1266 HIV-1 sequences available in the LANL repository. To assess HIV subtype LTR activity, HIV-LTR plasmid constructs were transfected into Jurkat cells using ViaFect (Promega), 24 h post transfection cells were treated with GSK2981278 (40 μM) for 24 h and luciferase activity measured as described above.

#### Chromatin immunoprecipitation (ChIP) and quantitative PCR

Jurkat cells were infected for 24 h with NL4.3-luc VSV-G (100 U RT/ 10^6^ cells) and then incubated in the presence of 10 μM GSK805 (or untreated control) for 24 h. U-2 OS cells were infected with NL4.3-luc VSV-G for 24 h, synchronised by serum shock and harvested 24h (CT0) or 36h (CT12) post synchronisation. Cells were fixed with 1% formaldehyde (Sigma) before quenching with 125 mM glycine. Cells were washed twice with cold PBS and lysed in SDS lysis buffer (10 mM Tris-HCl (pH 8.0), 10 mM NaCl, 1% NP-40) supplemented with a protease inhibitor cocktail (Roche Complete). Samples were diluted (1:1) with ChIP dilution buffer (0.01% SDS, 1.1% Triton, 0.2 mM EDTA; 16.7 mM Tris pH 8.1, 167 mM NaCl) and sonicated using a Bioruptor sonicator (30 min, 15 s on, 15 s off). Lysates were clarified by centrifugation and a small sample reserved as an input control in subsequent steps. Samples were precleared with Protein A agarose beads (Millipore), immunoprecipitated with anti-BMAL1 antibody (abcam, Ab3350), anti-REV-ERBα (NRIDI) antibody (Proteintech, 14506-I-AP), anti-RORC antibody (eBioscience, AFKJS-9) or rabbit IgG (Sigma, NI01) and precipitated with Protein A agarose beads. Samples were washed in low salt buffer (0.1% SDS, 1% Triton, 2 mM EDTA, 20 mM Tris pH 8.1, 150 mM NaCl), high salt buffer (0.1% SDS, 1% Triton, 2 mM EDTA, 20 mM Tris pH 8.1, 500 mM NaCl), LiCl Buffer (1% Igepal, 1 mM EDTA, 10 mM Tris pH 8.1, 250 mM LiCl, 1% sodium deoxycholate) and finally twice in TE wash buffer (10 mM Tris pH 8.0, 1 mM EDTA). Complexes were released from the beads using elution buffer (0.1 M NaHCO3, 1% SDS) and reverse crosslinked overnight at 65°C, shaking at 1400 rpm in the presence of 200 mM NaCl. After treatment with Proteinase K (Sigma) and RNaseA (Sigma), DNA was purified with MiniElute PCR Purification columns (Qiagen). qPCR was performed using a SYBR green qPCR mastermix (PCR Biosystems) in a LightCycler96 (Roche) with the primers listed above. The % input was calculated for each sample and normalised to IgG, allowing us to determine the fold enrichment of binding.

#### Flow cytometry

To measure cell viability, control or drug treated cells were stained with LIVE/Dead™ Fixable Aqua (Life Technologies) and fixed with 4% PFA (Santa Cruz) for 10 min at room temperature. Sample data was acquired on a Cyan ADP flow cytometer (Beckman Coulter) and data analyzed using FlowJo (TreeStar).

#### Bioinformatic analysis

The Eukaryotic Promoter Database^71^ was used to identify RORC binding motifs in circadian promoters. HIV-1 host factors were obtained from Hiatt et al.^34^ and cycling genes identified using the Circa Database^35^ by analysing the human datasets deposited on the platform. BMAL1 regulated genes were obtained from a published ChIP-seq dataset of mouse liver.^36^ REV-ERBα^37^ and RORC^38^ target genes were obtained from ChIP-seq datasets of mouse T cells. Promoter regions of the genes encoding HIV-1 host factors^34^ were inspected for the presence of E-box (CANNTG, canonical CACGTG) or RORE (RGGTCA) in sequences up to -1kb upstream of the TSS using HOMER (Hypergeometric Optimization of Motif EnRichment^39^). Gene ontology (GO) analysis was performed using ShinyGO,^40^ whereby each node represents an enriched GO term and related GO terms are connected by lines, whose thickness reflects the percentage of overlapping genes.

### Quantification and statistical analysis

Data was analysed using GraphPad Prism version 9.4.1. *p* values were determined using Mann-Whitney or Kruskal-Wallis tests. Significance values are indicated as ∗ *p* < 0.05, ∗∗ *p* < 0.01, ∗∗∗ *p* < 0.001, all data are presented as mean value ± SEM or mean value ± S.D. Please see individual figure legends for further details.

## Data Availability

The authors declare that all data supporting the findings of this study are available in the article along with supplemental information data.
