# Supplementary material for: Molecular components of the circadian clock regulate HIV-1 replication
Source: iScience. 2023 May 29;26(7):107007. doi: 10.1016/j.isci.2023.107007 (PMC10391662; doi:10.1016/j.isci.2023.107007)
Supplement: Document S1. Figures S1–S9 and Table S1 [file mmc1.pdf]

iScience, Volume *26*

Supplemental information

Molecular components of the circadian clock regulate HIV-1 replication

Helene Borrmann, Görkem Ulkar, Anna E. Kliszczak, Dini Ismed, Mirjam Schilling, Andrea Magri, James M. Harris, Peter Balfe, Sridhar Vasudevan, Persephone Borrow, Xiaodong Zhuang, and Jane A. McKeating

Supplementary Figure 1

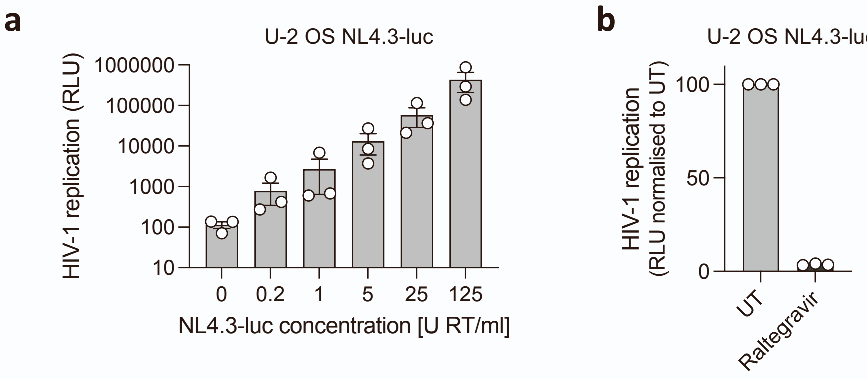

Supplementary Figure 1. U-2 OS cells support HIV-1 infection. (a) U-2 OS cells were infected with NL4.3-luc VSV-G for 24 h, cells lysed and luciferase activity (relative light units, RLU) measured as a read out for HIV-1 replication (mean ± S.E.M., n=3). **(b)** U-2 OS cells were pre-treated with the integrase inhibitor raltegravir (30 µM) for 24 h. Treated cells were infected with 100 U RT/ml NL4.3-luc VSV-G for 24 h in the presence of raltegravir and luciferase readings obtained 24 h post infection (mean ± S.E.M., n=3). (Related to Fig.1).

Supplementary Figure 2

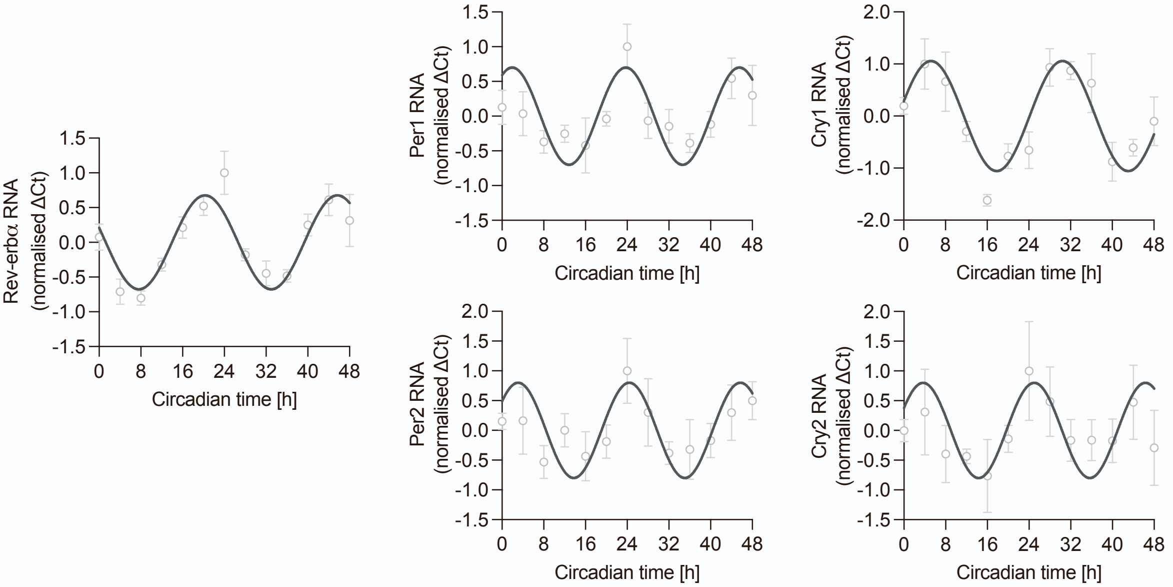

Supplementary Figure 2. Circadian gene expression in synchronised U-2 OS cells. U-2 OS cells were synchronised by serum shock for 1 h. 24h later, cells were harvested at 4 h intervals, RNA was extracted and expression of Rev-erbα, Per1, Per2, Cry1 and Cry2 RNAs relative to B2M housekeeper was measured by qPCR (mean ± S.E.M., n=4, normalised to peak). (Related to Fig.1).

Supplementary Figure 3

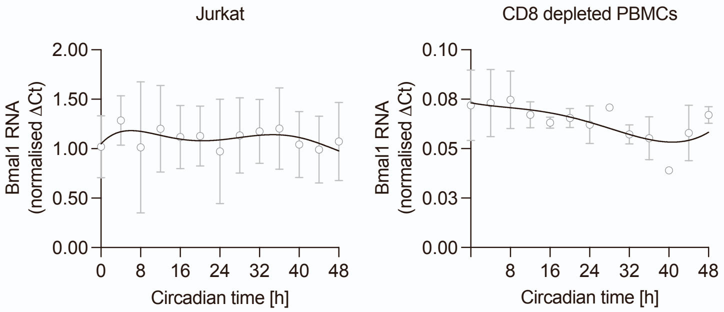

Supplementary Figure 3. Synchronisation of immune cells. CD8 depleted PBMCs were activated for 3 days with anti-CD3/CD28. Activated CD8 depleted PMBCs and Jurkat cells were synchronised by serum shock for 1 h and harvested in 4 h intervals for 48 h, starting 24 h post synchronisation. RNA was extracted, reverse transcribed and Bmal1 RNA relative to B2M housekeeper was quantified via qPCR (n=2, mean ± S.D). (Related to Fig.1).

Supplementary Figure 4

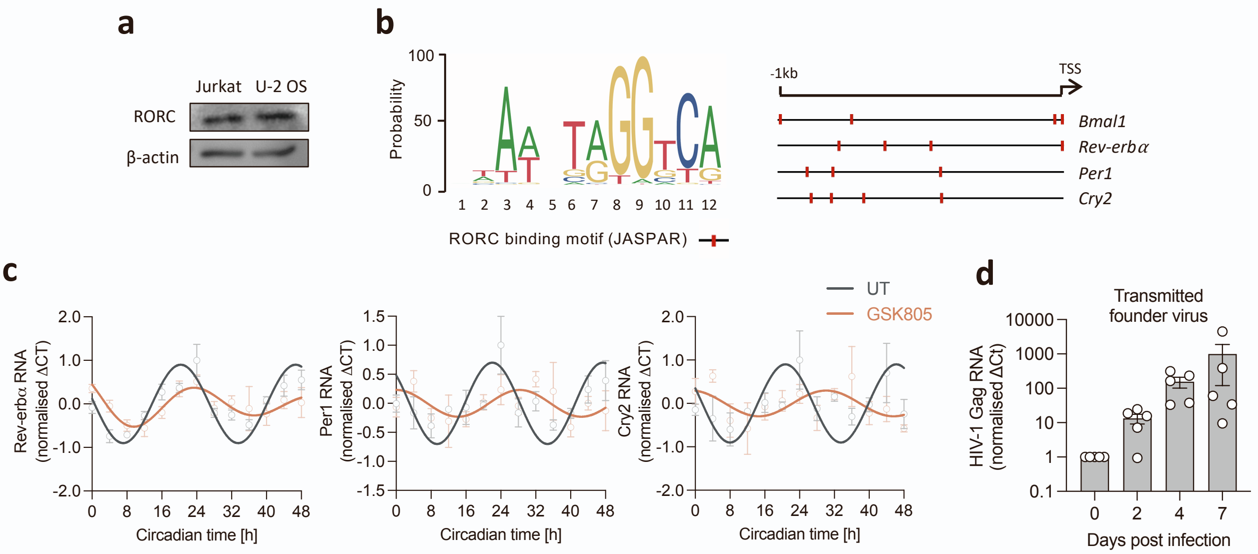

Supplementary Figure 4. GSK805 disturbs circadian gene expression in U-2 OS and primary T cells. (a) RORC expression in U-2 OS and Jurkat cell lysates (β-actin as control, representative of n=2). **(b)** The consensus sequence of the RORC DNA binding motif (JASPAR database) is shown, and the level of conservation is reflected by the height of the bases (y axis 0–100%). Presence and location of RORC motifs in *Bmal1, Rev-erbα, Per1* and *Cry2* promoter (1kb downstream of transcriptional start site (TSS), analysed with The Eukaryotic Promoter Database[S1]) is indicated by red symbols. **(c)** U-2 OS cells were synchronised by serum shock, treated with GSK805 (10 µM) and harvested at 4 h intervals, followed by RNA extraction and qPCR detection of RNA relative to a B2M housekeeper (mean ± S.E.M., n=3, normalised to peak). **(d)** CD8 depleted PBMCs were activated for 3 days with anti-CD3/CD28 and spinoculated with transmitted founder virus CH185 for 2 h. Cells were lysed, RNA extracted, DNA digested, reverse transcribed and HIV-1 Gag RNA measured by qPCR over 7 days (mean ± S.E.M., n=5; three of the biological repeats used cells pooled from three healthy donors, two further repeats were from a single HIV-seronegative donor each). (Related to Fig.3).

Supplementary Figure 5

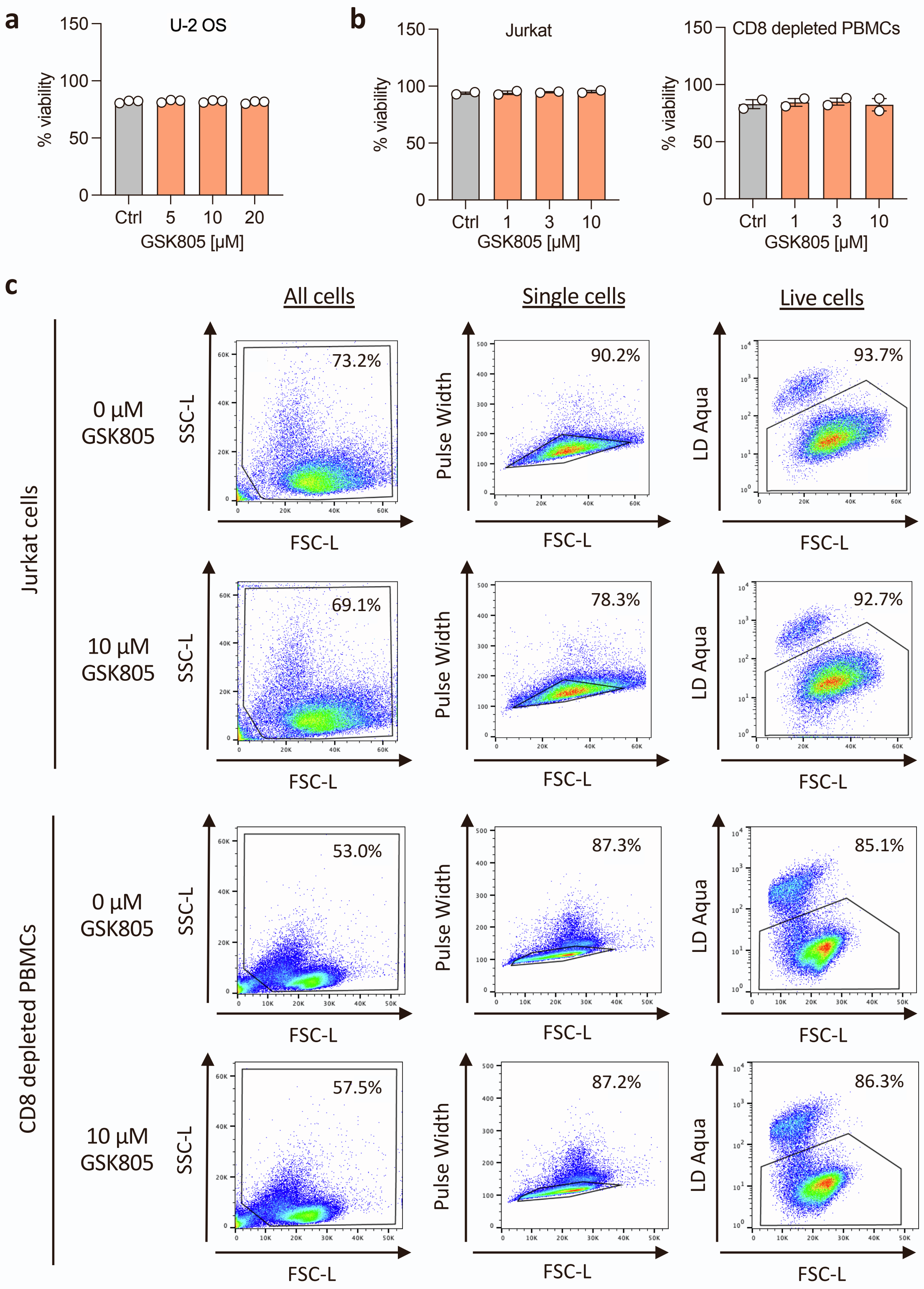

Supplementary Figure 5. Non-cytotoxicity of GSK805 for various cell types. (a) U-2 OS cells were treated with the RORC inverse agonist GSK805 at a range of doses for 24 h and cytotoxicity determined using an LDH assay. Data are expressed as % viability (mean ± S.E.M., n=3). **(b)** Jurkat cells or activated CD8 depleted PBMCs were treated with the ROR inverse agonist GSK805 for 24 h, and viability assessed by flow cytometry using an Aqua live-dead stain (mean + S.E.M., n=2). **(c)** Representative dot plots illustrating gating strategy and analysis. SSC = Side Scatter, FSC = Forward Scatter. (Related to Fig.3).

Supplementary Figure 6

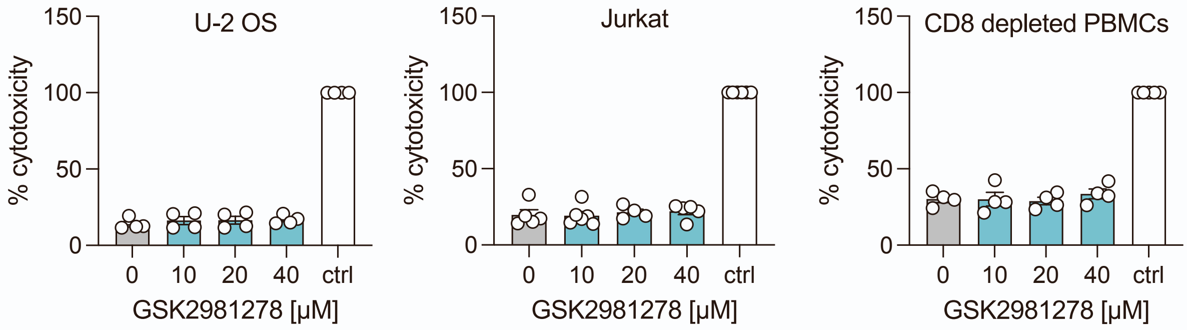

Supplementary Figure 6. Non-cytotoxicity of GSK2981278 for various cell types. U-2 OS, Jurkat or CD8 depleted PBMCs were treated with the ROR inverse agonist GSK2981278 at a range of doses for 24 h and cytotoxicity determined using a LDH assay (mean ± S.E.M., n=4-6). Data are expressed relative to the positive control representing total cell lysate (100% cytotoxicity). (Related to Fig.4).

Supplementary Figure 7

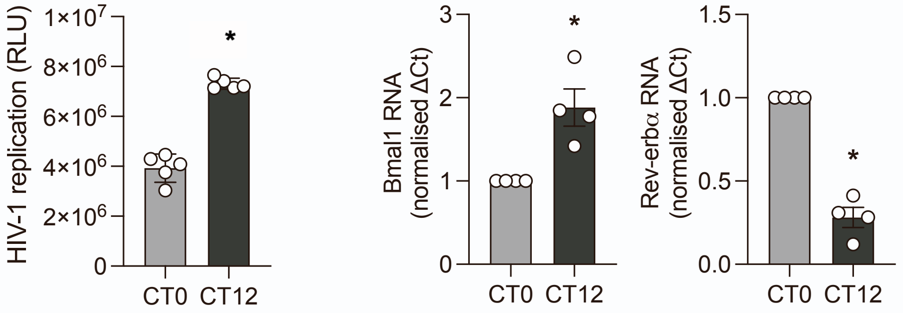

Supplementary Figure 7. Synchronisation data for U-2 OS ChIP. U-2 OS cells were infected with HIV-1 NL4.3-luc VSV-G for 24 h, synchronised by serum shock for 1 h, and cells were harvested 24 h (CT0) and 36 h (CT12) post synchronisation. Luciferase was measured as readout for viral replication (representative for n=4, ± S.D, Mann-Whitney test), RNA was extracted and expression of Bmal1 and Rev-erbα RNAs relative to B2M housekeeper was measured by qPCR (mean ± S.E.M., n=4, Mann-Whitney test). (Related to Fig.5).

Supplementary Figure 8

a

Cycling genes (39)

Dependency factors	Restriction factors
AFF1	AMBRA1
CBFB	ATP2A2
CCNT1	CEP57
CDK9	EIF2AK2
CNTNAP1	HDAC3
COPS8	HEXIM1
EIF3D	LDLR
EIF3E	MEPCE
EIF3H	PELO
GPS1	P4HB
HNRNPH3	RANBP2
MATR3	RBM17
MSH2	SDCCAG8
PARP4	SMG6
PELO	
PHB	
PREB	
PSIP1	
RNF126	
RUVBL1	
TCEB1	
TIMM13	
TMED9	
TOMM40	
USP11	
VDAC1	

b

BMAL1 targets (28)

Dependency factors	Restriction factors
PELO	AMBRA1
AFF1	ATP2A2
COPS4	CEP57
CXCR4	DDB1
EIF3E	EIF2AK2
HNRNPH3	G3BP1
HYOU1	LDLR
MATR3	MEPCE
MCM6	P4HB
NCAPD3	PELO
PHB	RBM17
PSMA1	
RNF126	
SARS	
SGPL1	
TOMM40	
VDAC1	
XPO5	

REV-ERBα targets (31)

Dependency factors	Restriction factors
AFF1	DDB1
CBFB	HEXIM1
CCNT1	HUWE1
CNTNAP1	ITGA4
COPS4	LDLR
CSDE1	NCOR1
CXCR4	RANBP2
DDX6	RARS
EIF3H	RBM17
ERP44	SDCCAG8
GLG1	SMG6
ITGAL	VPRBP
MATR3	
MCM6	
MSH2	
PSMA1	
SPCS3	
TIMM13	
VDAC1	

RORC targets (60)

Dependency factors	Restriction factors
AFF1	AMBRA1
ANKRD17	ATP2A2
CBFB	CEP57
CCNT1	COPS2
CHAF1B	DDB1
CHP1	G3BP1
CNTNAP1	HEXIM1
COPS3	ITGA4
COPS4	LDLR
COPS8	NCOR1
CSDE1	NDUFB10
CXCR4	RANBP2
DDX6	RBM17
EIF3E	SDCCAG8
EIF3H	SMG6
EIF3M	VPRBP
ERP44	
GLG1	
HGH1	
HYOU1	
ITGAL	
MATR3	
MCM6	
MOGS	
MSH2	
NCAPD3	
NUDC	
PHB	
PHB2	
PREB	
PSIP1	
PSMA1	
RUVBL1	
SARS	
SEC61A1	
SGPL1	
SPCS3	
STRAP	
TCEB1	
TIMM13	
TMED9	
TOMM40	
VDAC1	
XPO5	

c

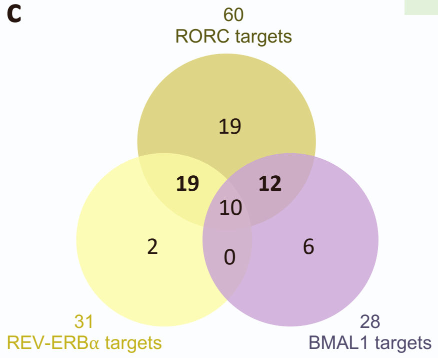

d

Encoding circadian motifs in promoter (40)

Canonical E-box

Dependency factors	Restriction factors
**EMC1**	DDB1
**HGH1**	EIF2AK2
HNRNPH3	**MEPCE**
PHB	
SKP1	
**SPCS3**	
**TIMM13**	

RORE

Dependency factors	Restriction factors
CCNT1	AMBRA1
CNTNAP1	ATP2A2
CSDE1	COPS2
CXCR4	HDAC3
DDX6	HEXIM1
EIF3E	ITGA4
EIF3M	LARP7
**EMC1**	**MEPCE**
ERP44	P4HB
GLG1	RARS
GPS1	
**HGH1**	
HIST1H3I	
HYOU1	
PREB	
PSMD7	
RANBP1	
SARS	
SGPL1	
SLIRP	
**SPCS3**	
STRAP	
**TIMM13**	
TOMM40	
UBA1	

Supplementary Figure 8. Cycling, BMAL1, REV-ERB and RORC regulated HIV-1 host factors. (a) HIV-1 related host proteins (dependency and restriction factors[S2]) were analysed for their rhythmic expression using the CircaDatabase[S3]. **(b)** BMAL1 regulated genes[S4], REV-ERBα regulated genes[S5] and RORC target genes[S6] were compared to factors known to regulate HIV-1 replication. **(c)** Overlap of BMAL1, REV-ERB and RORC target genes. **(d)** HOMER (Hypergeometric Optimization of Motif EnRichment tool) was used to analyse promoter regions (up to -1kb from TSS) of HIV-1 host factors[S2] and identified gene promoters encoding a canonical E-box motif 'CACGTG' or a ROR response element (RORE) 'RCGTCA'. Gene promoters that encode both E-box and RORE are written in bold. (Related to Fig.6).

Supplementary Figure 9

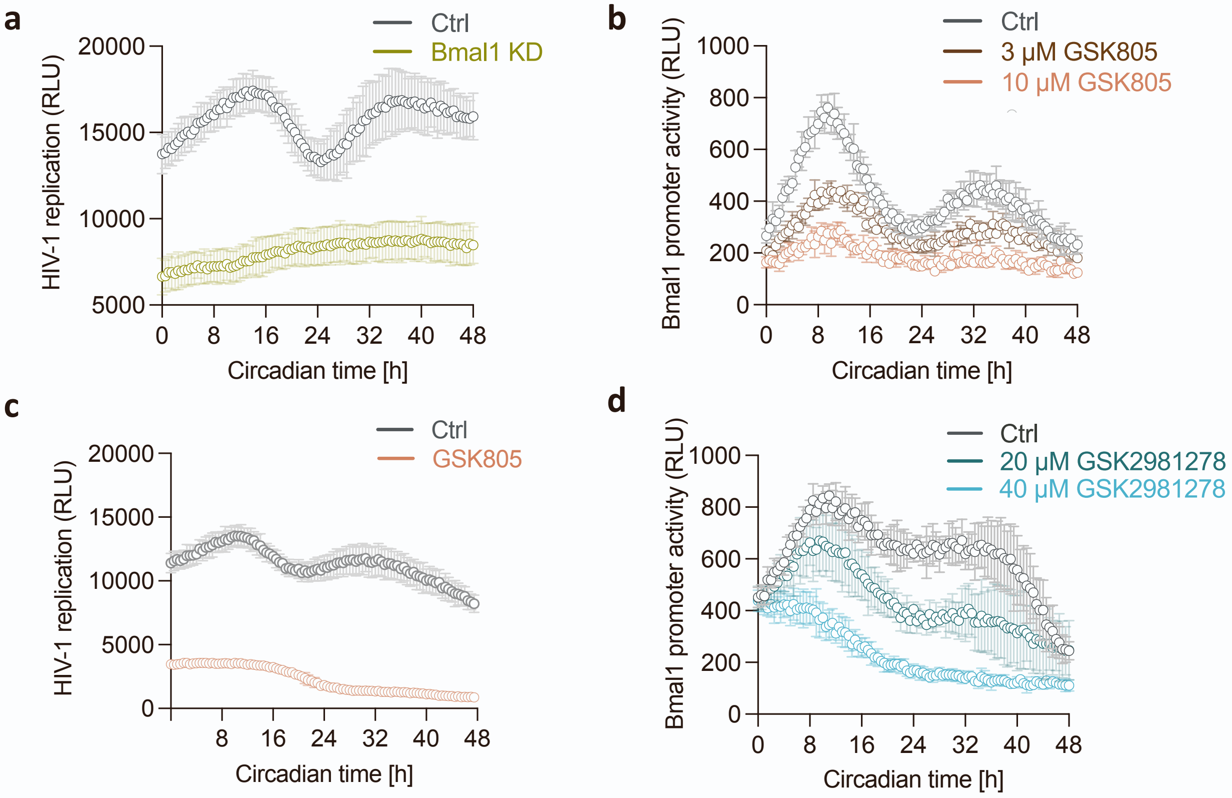

Supplementary Figure 9. Raw luminescence values for real time measurements. (a) U-2 OS parental control (ctrl) cells or U-2 OS Bmal1 knock-down (KD) cells generated by shRNA mediated silencing were infected with NL4.3-luc VSV-G, synchronised by serum shock and viral replication measured by luciferase readout every 30 min (representative of n=3, mean ± S.D., related to Figure 2a). **(b)** U-2 OS cells stably expressing luciferase under control of the Bmal1 promoter (Bmal1-luc) were synchronised, treated with 3 µM or 10 µM GSK805 (or untreated control) and luciferase measured at 30 min intervals (representative of n=3, mean ± S.D., related to Figure 3a). **(c)** U-2 OS cells infected with NL4.3-luc VSV-G were synchronised, treated with 10 µM GSK805 (or untreated control) and luciferase measured at 30 min intervals (representative of n=3, mean ± S.D., related to Figure 3d). **(d)** U-2 OS Bmal1-luc were synchronised, treated with 20 µM or 40 µM GSK2981278 (or untreated control) and luciferase measured at 30 min intervals (representative of n=4, mean ± S.D., related to Figure 4a).

Supplementary Table 1. Oligonucleotides, related to STAR methods.

Gene expression primers (human)

	Forward 5' – 3'	Reverse 5' – 3'
**Ambra1**	CTCTTCCTCAGACAACCAGGGT	TCCAAGCGAAGGTGCAGACATC
**Atp2A2**	GGACTTTGAAGGCGTGGATTGTG	CTCAGCAAGGACTGGTTTTCGG
**B2M**	CTACACTGAATTCACCCCCACTG	ACCTCCATGATGCTGCTTACATG
**Arntl (Bmal1)**	GCTCAGGAGAACCCAGGTTATC	GCATCTGCTTCCAAGAGGCTCA
**Ccnt1**	TGTTGACGCCACTGTGACCTTG	GTTTTCTTGGCAGCCTCGCATG
**Cntnap1**	GCCTTGTACTGCAACTGTGACG	CTCAGAAGTGGAGCGGTTCGTA
**Cops2**	CTGATGTGGAGAGCTTGCTGGT	GGTTGGTCCATTTATCTAGTGCAG
**Cry1**	GCAGTTGCTTGCTTCCTGACAC	GACAGCCACATCCAACTTCCAG
**Cry2**	AGGAGAACCACGACGAGACCTA	CCGTTCCAAGTGCTTATCCAGG
**Csde1**	GTAGTTTGTGCCATGAAGGAGGC	CCACATCATCGCCAGGCTGTAA
**Cxcr4**	CTCCTCTTTGTCATCACGCTTCC	GGATGAGGACACTGCTGTAGAG
**Ddb1**	CATTCCTCGCTCCATCCTGATG	CCTTCTTACGGTCGCTCAACAG
**Ddx6**	CCGAAATGGCTTATGCCGCAATC	GGAGATAGGTCTCTGCCAGCTT
**Eif2Ak2**	GAAGTGGACCTCTACGCTTTGG	TGATGCCATCCCGTAGGTCTGT
**Eif3E**	CTTTCTGTCGCATCCACCAGTG	TCAATCTTGGCATCCAGTCTTGC
**Eif3M**	GAGAACTCGGAAGGTGGACTTC	CCAGGATCAAGAGTAGGGATACC
**Emc1**	TGGTAACAGCCTCAGGCAAGCT	GAGCAGTAGTTCTCTGGACCATC
**Erp44**	AGTAGTGTTTGCCAGAGTTGATTG	CTGCCAATGCTTTCACTGATCGC
**Glg1**	GGTAGAAGACTGTGAACACCGTC	CACTGGTCTCATTCCAACCGTG
**Gps1**	ACTACTGCACCAGCGCCAAACA	ACTCAGCCTTGCTGACGTAGCT
**Hdac3**	GAGTTCTGCTCGCGTTACACAG	CGTTGACATAGCAGAAGCCAGAG
**Hexim1**	GAGGACAGTAGGTGGCAATCGA	AGGCAGCTAGATTCTGGACAGG
**Hgh1**	AGCCTACCTGATCCTTCGAGAG	GCAGGTTTTCCATGCCACGTTC
**Hist1H3I**	GGATACCAACCTGTGCGCCATT	GGACAGACTTCTTGGGCTGATAG
**Hnrnph3**	GGAGGTTTTGATGACTATGGTGG	CCTGAACTTGCATCACCAGCTC
**Hyou1**	CTTCAACCTGGATGAGAGTGGC	ACAGGCTGGAAATGGTGTTGCC
**Itga4**	GCATACAGGTGTCCAGCAGAGA	AGGACCAAGGTGGTAAGCAGCT
**Larp7**	AGAGCCTCTACCTGGCAGGAAA	ACTGCTTGAGCATCCTCAGGAG
**Mepce**	TGGAGCGGACACATCAGTCTTC	CCCAGTTCAGATGCACCCACTT
**P4Hb**	TCACCAAGGAGAACCTGCTGGA	GGCAAGAACAGCAGGATGTGAG
**Per1**	TCAACTGCCTGGACAGCATCCT	TCAGAGGCTGAGGAGGTGGTAT
**Per2**	AGCTGCTTGGACAGCGTCATCA	CCTTCCGCTTATCACTGGACCT
**Phb**	AAGCGGTGGAAGCCAAACAGGT	GCCAGTGAGTTGGCAATCAGCT
**Preb**	AAGTCGTCTCCTGCCTCGATGT	CACATCCGTCACCACAATGCCA
**Psmd7**	GATGTGAAGCCGAAGGACCTAG	TCCTCAGCTTCCTCTGCTCCAA
**Ranbp1**	ACCATGACCCTCAGTTTGAGCC	AGTGCCTCGCTCCTTCCATTCT
**Rars**	GAAACAGTGCGCCTCATGGATC	AGCCATACGCAACGGATGTCTG
**Nr1d1 (Rev-erbα)**	CTGCCAGCAATGTCGCTTCAAG	TGGCTGCTCAACTGGTTGTTGG
**Sars**	CCCAGAGAATGTGCTGAGTTTCG	CTCAAACCGCTCTGCTTCCAAC
**Sgpl1**	GAACACTGCCATGCTCGTCTGT	GATGAGGAAGCCTCCCAGACAA
**Skp1**	TCCTGAGGAGATTCGCAAGACC	ACTTCTCTTCACACCACTGGTTC
**Slirp**	TTCCTTGGACTGCGGCGTCGA	CCAAACCTCTGTGAAAGCCAGTC
**Spcs3**	GAGAGGTGATAATCCGAAGCTGC	TGGTACGACGTTCCAAGACAGG
**Strap**	GATGCCACCAAAGCAGCTACAG	CCTGCGTGAAATCCACAGTCTTG
**Timm13**	GAGCAGGTGAAAGTGCAGATCG	TGTAGCGGTCCATGCACATGGC
**Tomm40**	CGAAGTTTGTGAACTGGCAGGTG	AAGGCGTGATGCTCTGGAGGTA
**Uba1**	TCCTCACAGAGGACAAGTGCCT	CTTGAGCAGCTCACAGCCAATG

HIV-1 primers

	Forward 5' – 3'	Reverse 5' – 3'
**NL4.3 Gag**	CGAGAGCGTCGGTATTAAGC	CTGAAGGGATGGTTGTAGCTG
**CH185 TF Gag**	CGAGAGCGTCAGTATTAAGA	CTTAAGAGCTGATTGAAGCTG

ChIP primers

	Forward 5' – 3'	Reverse 5' – 3'
**HIV-LTR E-box 1&2**	ACCAGAGAGACCCAGTACAGGCA	GCTGGGGACTTTCCAGGGAGGT
**HIV-LTR E-box 3**	TAGTACTCCGGATGCAGCTCTCGG	AGCATGGGATGGAGGACCCGGA
**HIV-LTR E-box 4**	GCTGTTGTTCTCTCCTTCATTGGCC	CACACCAGGGCCAGGGATCAGA
**Per1 promoter E-box**	GTCAAGGAAAATCCCCAGCTTCTG	CCAAGATTGGTGACGTAAATGCCA
**HIV-LTR RORE**	TCCATGCAGGCTCACAGGGTGT	CACCAGGGCCAGGGGTCAGATA
**Bmal1 promoter RORE**	TTGGGCACAGCGATTGGT	GTAAACAGGCACCTCCGTCC

Supplementary References.

[S1] Dreos, R., Ambrosini, G., Périer, R.C., and Bucher, P. (2014). The Eukaryotic Promoter Database: expansion of EPDnew and new promoter analysis tools. Nucleic Acids Research *43*, D92-D96. 10.1093/nar/gku1111.

[S2] Hiatt, J., Hultquist, J.F., McGregor, M.J., Bouhaddou, M., Leenay, R.T., Simons, L.M., Young, J.M., Haas, P., Roth, T.L., Tobin, V., et al. (2022). A functional map of HIV-host interactions in primary human T cells. Nat Commun *13*, 1752. 10.1038/s41467-022-29346-w.

[S3] Pizarro, A., Hayer, K., Lahens, N.F., and Hogenesch, J.B. (2013). CircaDB: a database of mammalian circadian gene expression profiles. Nucleic Acids Res *41*, D1009-1013. 10.1093/nar/gks1161.

[S4] Beytebiere, J.R., Trott, A.J., Greenwell, B.J., Osborne, C.A., Vitet, H., Spence, J., Yoo, S.H., Chen, Z., Takahashi, J.S., Ghaffari, N., and Menet, J.S. (2019). Tissue-specific BMAL1 cistromes reveal that rhythmic transcription is associated with rhythmic enhancer-enhancer interactions. Genes Dev *33*, 294-309. 10.1101/gad.322198.118.

[S5] Chang, C., Loo, C.S., Zhao, X., Solt, L.A., Liang, Y., Bapat, S.P., Cho, H., Kamenecka, T.M., Leblanc, M., Atkins, A.R., et al. (2019). The nuclear receptor REV-ERBα modulates Th17 cell-mediated autoimmune disease. Proc Natl Acad Sci U S A *116*, 18528-18536. 10.1073/pnas.1907563116.

[S6] Ciofani, M., Madar, A., Galan, C., Sellars, M., Mace, K., Pauli, F., Agarwal, A., Huang, W., Parkhurst, C.N., Muratet, M., et al. (2012). A validated regulatory network for Th17 cell specification. Cell *151*, 289-303. 10.1016/j.cell.2012.09.016.
